# Seasonal dietary shifts enhance parasite transmission to lake salmonids during ice cover

**DOI:** 10.1002/ece3.6173

**Published:** 2020-04-08

**Authors:** Sebastian Prati, Eirik H. Henriksen, Rune Knudsen, Per‐Arne Amundsen

**Affiliations:** ^1^ Department of Arctic and Marine Biology Faculty of Biosciences, Fisheries and Economics UiT The Arctic University of Norway Tromsø Norway

**Keywords:** *Salmo trutta*, *Salvelinus alpinus*, seasonality, subarctic, winter

## Abstract

Changes in abiotic and biotic factors between seasons in subarctic lake systems are often profound, potentially affecting the community structure and population dynamics of parasites over the annual cycle. However, few winter studies exist and interactions between fish hosts and their parasites are typically confined to snapshot studies restricted to the summer season whereas host‐parasite dynamics during the ice‐covered period rarely have been explored. The present study addresses seasonal patterns in the infections of intestinal parasites and their association with the diet of sympatric living Arctic charr (*Salvelinus alpinus*) and brown trout (*Salmo trutta*) in Lake Takvatn, a subarctic lake in northern Norway. In total, 354 Arctic charr and 203 brown trout were sampled from the littoral habitat between June 2017 and May 2018. Six trophically transmitted intestinal parasite taxa were identified and quantified, and their seasonal variations were contrasted with dietary information from both stomachs and intestines of the fish. The winter period proved to be an important transmission window for parasites, with increased prevalence and intensity of amphipod‐transmitted parasites in Arctic charr and parasites transmitted through fish prey in brown trout. In Arctic charr, seasonal patterns in parasite infections resulted mainly from temporal changes in diet toward amphipods, whereas host body size and the utilization of fish prey were the main drivers in brown trout. The overall dynamics in the community structure of parasites chiefly mirrored the seasonal dietary shifts of their fish hosts.

## Graphical abstract

Seasonal variation in prevalence and intensity of trophically transmitted intestinal parasites hosted by Arctic charr (*Salvelinus alpinus*) and brown trout (*Salmo trutta*) in high‐latitude lakes is linked to dietary shifts occurring throughout the seasons. Winter proved to be an important transmission window, with increased prevalence and intensity of amphipod‐transmitted parasites in Arctic charr and parasites transmitted through piscivory in brown trout. In Arctic charr, seasonal patterns in intestinal parasite infections resulted mainly from temporal changes in diet towards amphipods, whereas host body size and the utilization of fish prey were the main drivers in brown trout.

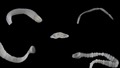

## 1 INTRODUCTION

Seasonal studies are important to understand the ecological dynamics of host‐parasite relationships. Like free‐living biota, parasite communities of both terrestrial and aquatic organisms vary seasonally due to temporal changes in abiotic and biotic factors (Altizer et al., 2006; Holmes, 1987, 1990; Kuhn, Knudsen, Kristoffersen, Primicerio, & Amundsen, 2016). Seasonal changes in the availability of intermediate hosts and infective stages may produce distinct seasonal patterns in parasite transmission (Esch & Fernández, 1993; Thieltges, Jensen, & Poulin, 2008), consequently affecting the parasite community structure and dynamics over the annual cycle. For instance, spring and summer, as opposed to winter, often correspond with a peak of helminth egg output and increased intensity of infections in wild Red deer populations from temperate regions (Albery et al., 2018). Such seasonal trends in parasite transmission are, however, not universal, as exemplified by the transmission of the nematode *Marshallagia marshalli* to reindeer in the high Arctic (Svalbard) which occurs throughout the winter months despite extreme cold conditions (Carlsson et al., 2012).

For freshwater fishes, seasonality in parasite transmission is documented from several host and parasite taxa (Chubb, 1979, 1980, 1982). However, few studies exist from lakes at high latitudes, where the changes between seasons are contrasting and profound. These changes are often dictated by the formation of ice cover, which can last for more than 6 months (Thompson, Ventura, & Camarero, 2009; Wrona et al., 2013). Studies of parasites in Finnish boreal lakes and coastal sea areas with similar ice‐cover duration have demonstrated the existence of host‐parasite dynamics during winter in various fish hosts (Karvonen, Cheng, & Valtonen, 2005; Valtonen & Crompton, 1990; Valtonen, Prost, & Rahkonen, 1990). The winter period has, however, traditionally been viewed as an insignificant season of low ecological importance in ice‐bound lakes (Salonen, Leppäranta, Viljanen, & Gulati, 2009), and the majority of host‐parasite studies in northern lake systems have addressed the ice‐free period (Knudsen, Klemetsen, & Staldvik, 1996; Tedla & Fernando, 1969). This bias toward summer and autumn studies may underestimate the importance of the winter as a transmission window for parasites in high‐latitude lakes.

The exposure to trophically transmitted parasites is often related to the abundances of intermediate and final hosts in the environment (Hechinger & Lafferty, 2005; Stutz, Lau, & Bolnick, 2014). Moreover, intra‐ and interspecific differences in exposure might arise from differences in feeding preferences, host interactions, and host‐parasite compatibility (Carney & Dick, 2000; Fernández, Brugni, Viozzi, & Semenas, 2010; Knudsen, Amundsen, Nilsen, Kristoffersen, & Klemetsen, 2008; Knudsen, Curtis, & Kristoffersen, 2004; Lagrue, Kelly, Hicks, & Poulin, 2011). For instance, Stutz et al. (2014) observed that within populations of three‐spined stickleback (*Gasterosteus aculeatus*) inhabiting the same habitat, infections of the benthic nematode *Eustrongylides* sp. were higher in fish consuming benthic prey while the copepod‐transmitted *Schistocephalus solidus* increased in fish consuming limnetic prey. Similarly, Grunberg, Brianik, Lovy, and Sukhdeo (2019) observed that anadromous variants of the alewife (*Alosa pseudoharengus*) were infected with more abundant and diverse parasite assemblages compared to a resident variant because they consumed a wider variety of prey from the diverse habitats used during their life cycle.

Beside the trophic behavior of the hosts, the availability of potential intermediate prey hosts is often regulated by seasonal changes in environmental factors, which can also affect the viability of free‐living parasite stages and thereby alter parasite transmission rates to fish (Chubb, 1982; Pietrock & Marcogliese, 2003). The structure of parasite communities in fish may consequently change seasonally with different prey and parasite species dominating in different seasons (Kennedy, 1997). Accounting for such seasonality in parasite transmission is important when conducting larger ecological studies (e.g., food‐web analyses) that include parasites.

In the present study, we address seasonal patterns in the intestinal parasite community of sympatric Arctic charr (*Salvelinus alpinus*) and brown trout (*Salmo trutta*) in subarctic Lake Takvatn, northern Norway, which is ice covered for approximately half the year. Lake Takvatn has been the subject of numerous ecological studies, including fish and food‐web analyses that comprise parasites (see Amundsen & Knudsen, 2009; Amundsen et al., 2013; Amundsen et al., 2019), thus constituting a good basis for seasonal studies addressing intestinal parasites. The intestinal parasites of Arctic charr and brown trout are typically transmitted trophically with diet as an important predictor of their community composition (Curtis, Bérubé, & Stenzel, 1995; Knudsen et al., 2008; Kuhn, Knudsen, et al., 2016).

Sympatric Arctic charr and brown trout segregate their diets during the ice‐free season with the former being the most generalist feeder (Eloranta, Knudsen, & Amundsen, 2013), which seems to drive differences in their parasite communities (Knudsen et al., 2008). The most common intermediate hosts for fish intestinal parasites in northern lakes include zooplankton (mainly copepods), amphipods, insect larvae, and fish. Typically, Arctic charr have more parasites transmitted via copepods than brown trout, which are more frequently parasitized via the consumption of amphipods, insect larvae, and fish as intermediate hosts (Henriksen et al., 2016; Knudsen et al., 2008; Paterson, Nefjodova, Salis, & Knudsen, 2019). However, under ice cover, both Arctic charr and brown trout feed mainly on benthic macroinvertebrates including amphipods and insect larvae in addition to increased piscivory in brown trout (Amundsen & Knudsen, 2009; Klemetsen, Amundsen, et al., 2003). Such seasonal changes in feeding ecology may be crucial for the structuring of parasite communities in salmonid hosts.

Here, we explore seasonal patterns in the infections of intestinal parasites and their association with the diet of Arctic charr and brown trout in Lake Takvatn. Firstly, we hypothesized that Arctic charr have a higher parasite diversity, prevalence, and abundance than brown trout throughout all seasons due to a broader dietary niche. We secondly hypothesized that Arctic charr will have increased infections (prevalence and intensity) of amphipod‐transmitted parasites while brown trout will aggregate more parasites transmitted via amphipods and fish prey during the winter period. Thirdly, we hypothesized that diet drives seasonal changes in the intestinal parasite communities. Consequently, the accumulation of parasites during the winter period will be particularly important for the overall differences in parasite community seen between the two fish host species.

## 2 MATERIALS AND METHODS

### 2.1 Study site

The study was conducted in Lake Takvatn, a dimictic and oligotrophic subarctic lake located 215 m above mean sea level in Troms county, northern Norway. The lake has a surface area of 15.2 km^2^ and a maximum depth of 88 m. Secchi depths range between 14 and 17 m, and phosphorous levels do not exceed five micrograms per liter (Eloranta et al., 2013). The lake is usually ice covered from late November to early June (Amundsen, Knudsen, & Klemetsen, 2007; Klemetsen, Knudsen, Staldvik, & Amundsen, 2003). In the winter of 2017–2018, the lake surface froze during the last week of November and the ice melted at the end of May. The maximum measured ice thickness was 100 cm in March. The temperatures observed in the upper water level (1 m depth) during the field sampling periods ranged between 12.8°C in summer (August) and 1.15°C under ice cover in winter (January). The only fish species present in the lake are brown trout, Arctic charr, and three‐spined stickleback (*G. aculeatus*) (see Amundsen and Knudsen (2009) for further details about the lake).

### 2.2 Fish sampling and processing

In total, 354 Arctic charr and 203 brown trout were sampled from the littoral habitat (<15 m depth) between June 2017 and May 2018 using multi‐meshed gillnets with panels of eight different mesh sizes from 10 to 45 mm, knot to knot (Table 1). The sampling was carried out monthly during the ice‐free season (June to November) and every second month during the ice‐covered period (December to May). During the ice‐covered period, gill nets were pulled out and retrieved through holes in the ice by means of submerged ropes. The ropes were positioned in the lake in December when the ice thickness was still modest. The nets were left in the lake overnight for approximately 12 hr during the ice‐free period and approximately 16 hr during the ice‐covered period. In the field, fork length in mm, weight, sex, and gonad maturation of all fish was recorded. Stomachs were opened, and the fullness degree was determined on a scale from 0% to 100%. Prey types were identified, and their contribution to the total stomach contents was calculated according to the method described by Amundsen (1995). The stomach contents were preserved in 96% alcohol, and the intestines were frozen to preserve the content, allowing subsequent parasitological and dietary analyses in the laboratory.

**Table 1:** Number and average fork length (in mm) ± *SD* of sampled fish individuals throughout the sampling period. No trout were captured in June 2017

Month	Season	Arctic charr		Brown trout	
		*N*	Mean with *SD*	*N*	Mean with *SD*
June 2017	Summer	50	304.7 mm ± 41.9	–	–
August 2018	Summer	50	232.9 mm ± 81.6	50	204.9 mm ± 42.0
September 2018	Autumn	24	316.5 mm ± 50.0	36	274.0 mm ± 72.3
October 2018	Autumn	36	277.5 mm ± 87.7	48	245.3 mm ± 105.7
November 2018	Early winter	50	243.3 mm ± 54.2	41	247.6 mm ± 99.8
January 2018	Early winter	50	273.8 mm ± 60.5	11	352.9 mm ± 122.5
March 2018	Late winter	50	256.3 mm ± 60.7	7	459.6 mm ± 125.1
May 2018	Late winter	44	261.9 mm ± 73.7	10	379.8 mm ± 110.1

### 2.3 Parasite sampling

**Figure 1:**
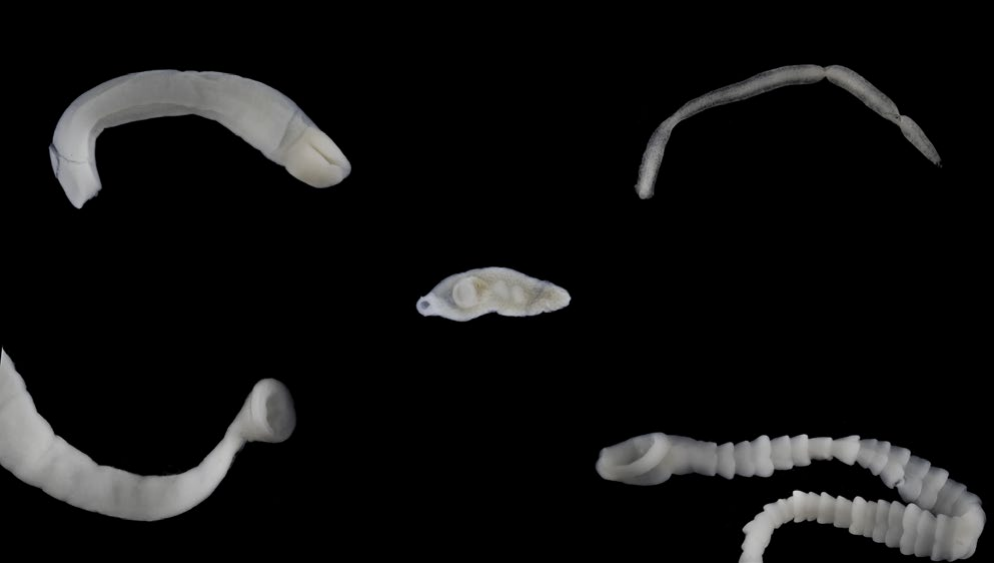
Examples of intestinal parasites of Arctic charr and brown trout: *Dibothriocephalus* sp. (upper‐left corner), *C. truncatus* (lower‐left corner), *Crepidostomum* sp. (center), *Proteocephalus* sp. (upper‐right corner), and *E. salvelini* (lower‐right corner)

The intestinal parasites were sampled by cutting the intestines open and sieving the contents including that of the pyloric caeca under running water with a 120‐micron mesh size nylon net. The collected material was then placed into a Petri dish with a physiological saltwater solution (9% NaCl). We found 5 taxa: *Crepidostomum* spp., *Cyathocephalus truncatus*, *Eubothrium salvelini*, *E. crassum,* and *Proteocephalus* sp., which use Arctic charr or brown trout as their final host. (Table 2, Figure 1). At least four potentially different species belonging to the genus *Crepidostomum* are found in Lake Takvatn (Soldánová et al., 2017), here grouped as *Crepidostomum* spp. as they are only distinguishable via genetic analysis. The only representative of the genus *Proteocephalus* is here described as *Proteocephalus* sp. since the exact species is not known. Additionally, the larval stage (plerocercoids) of two different species of *Dibothriocephalus* (formerly *Diphyllobothrium* (Waeschenbach, Brabec, Scholz, Littlewood, & Kuchta, 2017)) were also recorded in the intestines of both Arctic charr and brown trout and are here grouped together as *Dibothriocephalus* spp. (Figure 1). The *Dibothriocephalus* spp. plerocercoids analyzed in this study include only the unencysted larvae found in the intestine, not those encysted in the viscera. The presence of unencysted plerocercoids in the intestine has previously been considered accidental. However, a high correlation between the number of unencysted plerocercoids and the degree of piscivory (see later), particularly in brown trout, strongly indicates that their presence was the result of recent ingestion of infected fish prey, and the *Dibothriocephalus* spp. plerocercoids were therefore taken into consideration for the analyses.

**Table 2:** Parasites found in the intestine of Arctic charr and brown trout and their life cycle

Parasite taxa	Stage	Intermediate hosts	Final hosts	Lifetime in the host
*Crepidostomum* spp. (trematode)	Adult	Amphipods/insect larvae	Arctic charr and brown trout	1 year (Thomas, 1958)
*Cyathocephalus truncatus* (cestode)	Adult	Amphipods	Arctic charr and brown trout	20–55 days (Okaka, 1984)
*Eubothrium salvelini* (cestode)	Adult	Copepods/fish	Arctic charr	1–2 year (Hanzelová et al., 2002)
*E. crassum* (cestode)	Adult	Copepods/fish	Brown trout	1–2 year (Hanzelová et al., 2002)
*Proteocephalus* sp. (cestode)	Adult	Copepods/fish	Arctic charr and brown trout	1 year (Scholz, 1999)
*Dibothriocephalus* spp. (cestode)	Larvae	Copepods/fish	Birds	Not known for unencysted plerocercoids

### 2.4 Prey types in the gastrointestinal tract

Only amphipods, insect larvae, zooplankton, and fish were considered for the stomach‐parasite analysis, as they are the potential intermediate hosts of the identified intestinal parasites. The importance of these prey in the fish was expressed as frequency of occurrence (Amundsen & Sánchez‐Hernández, 2019). The dietary information from the individual stomach samples was incomplete due to a high number of empty stomachs (Arctic charr *N* = 115, brown trout *N* = 29), especially during winter‐time. To overcome this issue, the intestinal contents of each fish were carefully examined for the presence of identifiable prey remains. The frequency of occurrence of prey types in the present study is therefore a combination of stomach and intestinal observations (i.e., the whole gastrointestinal tract) of each individual fish. The implementation of the intestinal prey data covered the missing diet information for 40% of the empty stomachs.

### 2.5 Statistical analysis

Descriptive and statistical analyses were performed with the open‐source software Rstudio (version 1.1.423, Rstudio Inc.) and QPweb (version 1.0.14, Reiczigel, Marozzi, Fábián, & Rózsa, 2019), both based in R (version 3.5.1, R Core Team, 2018). To investigate differences in parasite load between Arctic charr and brown trout, five quantitative parameters (mean number of taxa, abundance, prevalence, intensity, and mean intensity) were analyzed according to Bush, Lafferty, Lotz, and Shostak (1997) and Poulin (1998). Mean number of taxa is defined as the mean number of parasite taxa per host individual. Mean number of parasite taxa was used instead of observed parasite species richness, as no seasonal differences between Arctic charr and brown trout were detected using the Jackknife method (Zelmer & Esch, 1999) as an estimator of parasite richness. The mean number of parasite taxa was compared between Arctic charr and brown trout using the Mann–Whitney *U* test. Abundance is the number of parasite individuals of a particular species in a single host species (infected and uninfected). Prevalence is defined as the proportion of host individuals infected by a particular parasite among the examined sample of a specific host species, usually expressed in percentage. Prevalence was compared between host species using a *χ* ^2^ test with Yates correction for each parasite taxa separately. Intensity is the number of parasite individuals of a particular species in a single infected host species. Mean intensity represents the average number of parasite individuals belonging to a particular species found in all hosts infected by that parasite (uninfected hosts excluded). To test for differences in mean intensity of each parasite taxa separately a nonparametric maximum test that combines Brunner–Munzel and Welch *U* tests was used as suggested by Welz, Ruxton, and Neuhäuser (2018).

To analyze seasonal variations in the infections of intestinal parasites, monthly data were merged into four seasonal periods to cope with the low winter sample size (Table 1). As the length of both Arctic charr and brown trout significantly differed among sampling seasons (One‐way ANOVA, *F*(3) = 3.844, *p* = .01 and *F*(3) = 21.78, *p* < .001, respectively), any size effect on the seasonal variation in parasite infections was also tested using a negative binomial generalized linear model (GLM) with length as a covariate. Negative binomial GLM is best suited to model the overdispersion of parasites distributions among hosts which is typically aggregated (Lindén & Mäntyniemi, 2011; Paterson & Lello, 2003; Rózsa, Reiczigel, & Majoros, 2000; Wilson & Grenfell, 1997). The model included parasite counts of infected hosts (i.e., intensity) as the response variable with seasons and fish length as predictors. The use of sex as a covariate did not produce significant results, and age was excluded from the analysis because part of the age data material was missing. The function glm.nb from the MASS package in R was used to run the model, and ANOVA (Type II) function from the Car package in R was adopted to assess the main effects. Similarly, to account for fish body size, seasonality in prevalence was tested using a binomial GLM.

To assess differences in parasite communities between seasons and host species, we used PERMANOVA (function Adonis in vegan package) on Bray–Curtis abundances matrices, thereafter, illustrating the results using nonmetric multidimensional scaling (NMDS). Canonical correspondence analysis (CCA) was used to assess the relationship between the abundance of parasite taxa (response variable), presence‐absence of prey types, and fish body length (explanatory variables). ANOVA‐like permutations (999 cycles, function ANOVA.cca in Vegan package) were used to test which variables explained a significant part of the variation in parasite abundance. Fish with no intestinal parasite infection were by default omitted from the CCA analyses. Species diversity across seasons was calculated using Shannon index (H′). Shannon index values of Arctic charr and brown trout were then compared with Hutcheson *t* test. A correlation matrix with the Winsorized correlation coefficient (Wilcox, 2001) was further used to analyze potential correlations between parasite prevalence and frequency of occurrence of prey types. This method was preferred over the widely used Spearman's rank and Kendall's tau correlation coefficients as it is more robust to distribution shape, sample size, and outliers (Tuğran, Kocak, Mirtagioğlu, Yiğit, & Mendes, 2015; Wilcox, 2001).

## 3 RESULTS

### 3.1 The intestinal parasite communities of Arctic charr and brown trout

**Figure 2:**
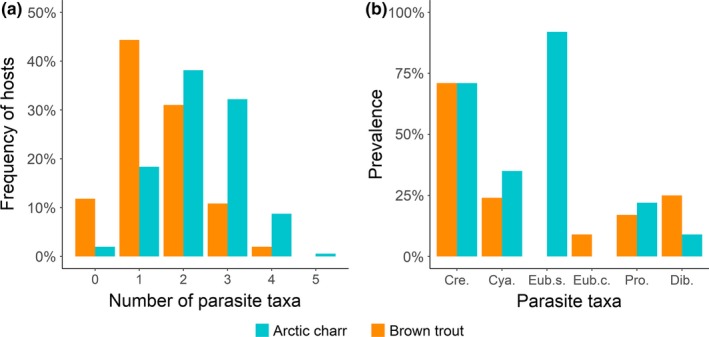
(a) Frequency distribution of the number of intestinal parasites taxa and (b) their prevalence in Arctic charr and brown trout (Cre. = *Crepidostomum* spp., Cya. = *C. truncatus,* Eub.s. = *E. salvelini*, Eub.c. = *E. crassum*, Pro. = *Proteocephalus* sp., and Dib. = *Dibothriocephalus* spp.)

Of all Arctic charr examined, 98% were infected with at least one intestinal parasite taxon and the mean number of parasite taxa per fish was 2.3 (±0.95 *SD*, Figure 2a). In total, five intestinal parasites taxa were recorded in Arctic charr (Figure 2b). Of these, the most common were *E. salvelini* with 92% and *Crepidostomum* spp. with 71% prevalence, whereas *C. truncatus*, *Proteocephalus* sp., and *Dibothriocephalus* spp. had much lower prevalence (<40%).

Of all brown trout examined, 88% were infected with at least one intestinal parasite taxon and the mean number of parasite taxa per fish was 1.5 (±0.91 *SD*), which was significantly less than in Arctic charr (Mann–Whitney *U* test, W = 52,280, *p* < .01**;** Figure 2a). Overall, five intestinal parasite taxa were found. The most common parasite was *Crepidostomum* spp. with a prevalence of 71%, whereas *E. crassum*, *C. truncatus*, *Proteocephalus* sp., and *Dibothriocephalus* spp. were less common (prevalence < 25%; Figure 2b). The prevalence of *Proteocephalus* sp. and *Dibothriocephalus* spp. differed significantly between Arctic charr and brown trout (*χ* ^2^(3) = 30.489, *p* < .001, and *χ* ^2^(3) = 8.021, *p* = .046, respectively), and in mean intensity only for *Crepidostomum* spp. (maximum test, *p* < .029). The prevalence and mean intensity of *C. truncatus* did not differ between Arctic charr and brown trout (all *p* > .05).

### 3.2 Seasonal variations of intestinal parasites in Arctic charr and brown trout

**Figure 3:**
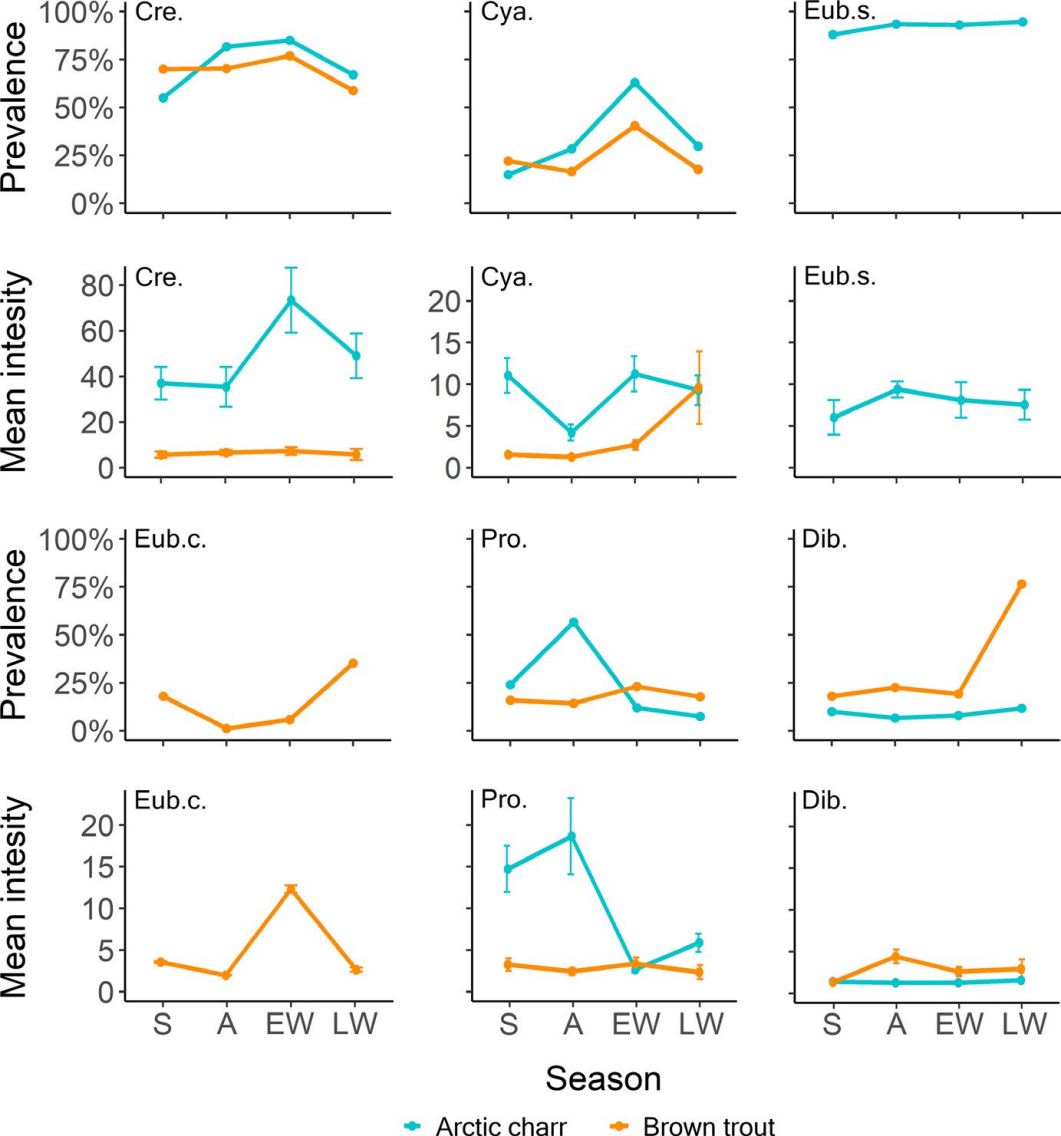
Prevalence and mean intensity (with 95% confidence intervals) of intestinal parasites in Arctic charr and brown trout throughout the main seasons (S = summer, A = autumn, EW = early winter, LW = late winter, Cre. = *Crepidostomum* spp., Cya. = *C. truncatus,* Eub.s. = *E. salvelini*, Eub.c. = *E. crassum*, Pro. = *Proteocephalus* sp., and Dib. = *Dibothriocephalus* spp.)

Both Arctic charr and brown trout displayed significant seasonal variations in the prevalence and intensity of several intestinal parasites (Table 3a,b, Figure 3). Parasite infections in the two salmonids were strongly influenced by both seasons and fish length (Table 3a,b). There were seasonal differences in prevalence and intensity for *Crepidostomum* spp., *C. truncatus,* and *Proteocephalus* sp. hosted by Arctic charr (all *p* < .05; Table 3b), and for *E. crassum*, *C. truncatus,* and *Proteocephalus sp.* (only prevalence) in brown trout (all *p* < .05; Table 3b).

**Table 3:** (a) Statistical result on variables associated with parasite intensity (ANOVA from GLM negative binomial regression) and (b) prevalence (ANOVA from GLM binomial regression) in Arctic charr and brown trout.

Parasite taxa	Variable	Arctic charr			Brown trout		
		*χ* ^2^	*d*ƒ	*p*	*χ* ^2^	*df*	*p*
(a) Parasite intensity							
*Crepidostomum* spp.	Season	29.97	3	<.001	2.28	3	.516
	Length	36.56	1	<.001	0.08	1	.771
*Cyathocephalus truncates*	Season	35.66	3	<.001	27.3	3	<.001
	Length	27.62	1	<.001	5.29	1	.021
*Eubothrium salvelini*	Season	8.87	3	.031	–	–	–
	Length	15.97	1	<.001	–	–	–
*E. crassum*	Season	–	–	–	15.23	3	.002
	Length	–	–	–	2.76	1	.096
*Proteocephalus* sp.	Season	61.10	3	<.001	7.47	3	.058
	Length	8.25	1	.004	20.82	1	<.001
*Dibothriocephalus* spp.	Season	3.74	3	.291	2.24	3	.525
	Length	7.59	1	.006	56.69	1	<.001
(b) Parasite prevalence							
*Crepidostomum* spp.	Season	28.20	3	<.001	2.05	3	.562
	Length	32.47	1	<.001	0.01	1	.913
*Cyathocephalus truncates*	Season	57.34	3	<.001	11.74	3	.008
	Length	3.905	1	.048	7.60	1	.006
*Eubothrium salvelini*	Season	3.18	3	.364	–	–	–
	Length	0.37	1	.544	–	–	–
*E. crassum*	Season	–	–	–	21.35	3	<.001
	Length	–	–	–	1.40	1	.237
*Proteocephalus* sp.	Season	53.18	3	<.001	9.29	3	.025
	Length	0.01	1	.943	30.66	1	<.001
*Dibothriocephalus* spp.	Season	2.11	3	.550	7.46	3	.059
	Length	4.31	1	.038	24.76	1	<.001

**Figure 4:**
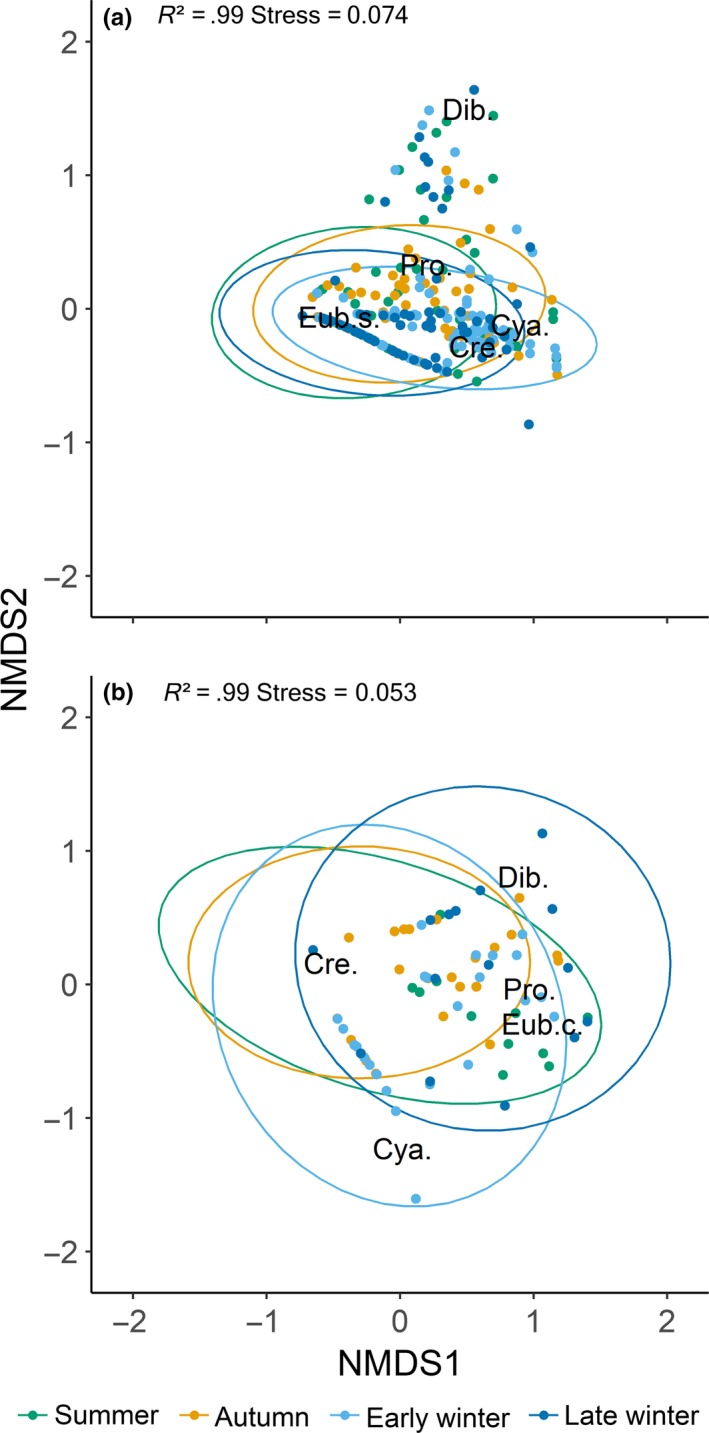
Nonmetric multidimensional scaling (NMDS) plot on Bray–Curtis distances of (a) Arctic charr and (b) brown trout showing dissimilarity in parasite community composition between seasons including 95% confidence intervals ellipses (Cre. = *Crepidostomum* spp., Cya. = *C. truncatus*, Eub.s. = *E. salvelini*, Eub.c. = *E. crassum*, Pro. = *Proteocephalus* sp., and Dib. = *Dibothriocephalus* spp.). NMDS converged on a three‐dimensional solution with an acceptable stress level

Seasonal differences in the intestinal parasite communities of Arctic charr and brown trout were also reflected by Bray–Curtis based NMDS plots (Figure 4a,b). In Arctic charr, the abundance of *C. truncatus*, *Crepidostomum* spp., and *E. salvelini* was clearly linked to the early and late winter period while that of *Proteocephalus* sp. with the autumn period. The abundance of *Dibothriocephalus* spp., on the contrary, was not related to any of the four sampling seasons (Figure 4a). Overall, dissimilarity in parasites abundance among seasons was significant (PERMANOVA, *F* = 0.059, *p* < .001). Similarly, in brown trout, seasonal differences in parasite abundance were significant (PERMANOVA, *F* = 0.051, *p* < .001) with *C. truncatus* and *Crepidostomum* spp. being more abundant in early winter, and *Dibothriocephalus* spp., *E. crassum,* and *Proteocephalus* sp. in late winter (Figure 4b).

There were seasonal differences in parasite diversity for both fish species. Arctic charr had the highest parasite diversity in autumn (H′ = 1.05) and the lowest in early winter (H′ = 0.64), while diversity in brown trout was at the minimum in autumn (H′ = 0.82) and peaked in late winter (H′ = 1.43). Discrepancies in parasite diversity between the two host species were overall significant (Hutcheson *t* test: *T* = 9.170, *p* < .001), and more pronounced during the ice‐covered period (Hutcheson *t* test: *T* = 16.164, *p* < .001) than in the ice‐free period (Hutcheson *t* test: *T* = 2.145 *p* = .032). Differences in parasite diversity between the ice‐free period and the ice‐covered period were significant in both host species and more pronounced in Arctic charr (Hutcheson *t* test: *T* = 26.175, *p* < .001) than in brown trout (Hutcheson *t* test: *T* = 6.064, *p* < .001).

### 3.3 Seasonal variations in diet

**Figure 5:**
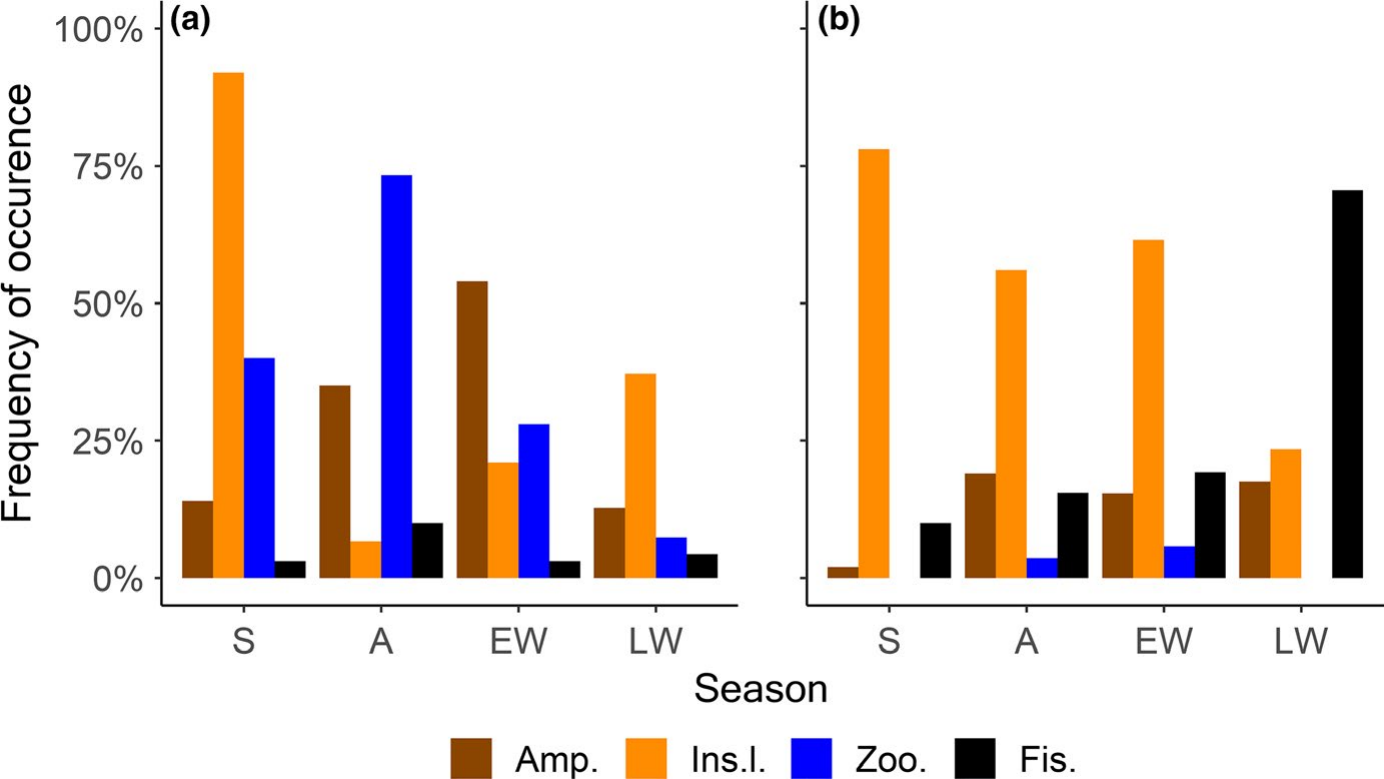
Seasonal variations in the frequency of occurrence of prey categories in the diet of Arctic charr (a) and brown trout (b) (S = summer, A = autumn, EW = early winter, LW = late winter, Amp. = amphipods, Ins.l. = insects larvae, Zoo. = zooplankton, and Fis. = fish). Prey categories not related to intestinal parasite transmission are excluded

Insect larvae, zooplankton, and amphipods were the most common overall prey for Arctic charr (Figure 5a). Insect larvae were the most common prey during late winter and summer, whereas zooplankton was the most common prey during autumn and amphipods during early winter. Fish prey had in contrast just a minor contribution to the diet of Arctic charr in all seasons. In brown trout, insect larvae and fish (mainly three‐spined stickleback) were the most important prey groups (Figure 5b). Insect larvae were the dominant prey from summer to early winter, while fish were the main prey in late winter. The occurrence of amphipods in the brown trout diet was relatively modest throughout all seasons, whereas zooplankton was recorded only in autumn and early winter.

### 3.4 Associations between parasites and diet

**Figure 6:**
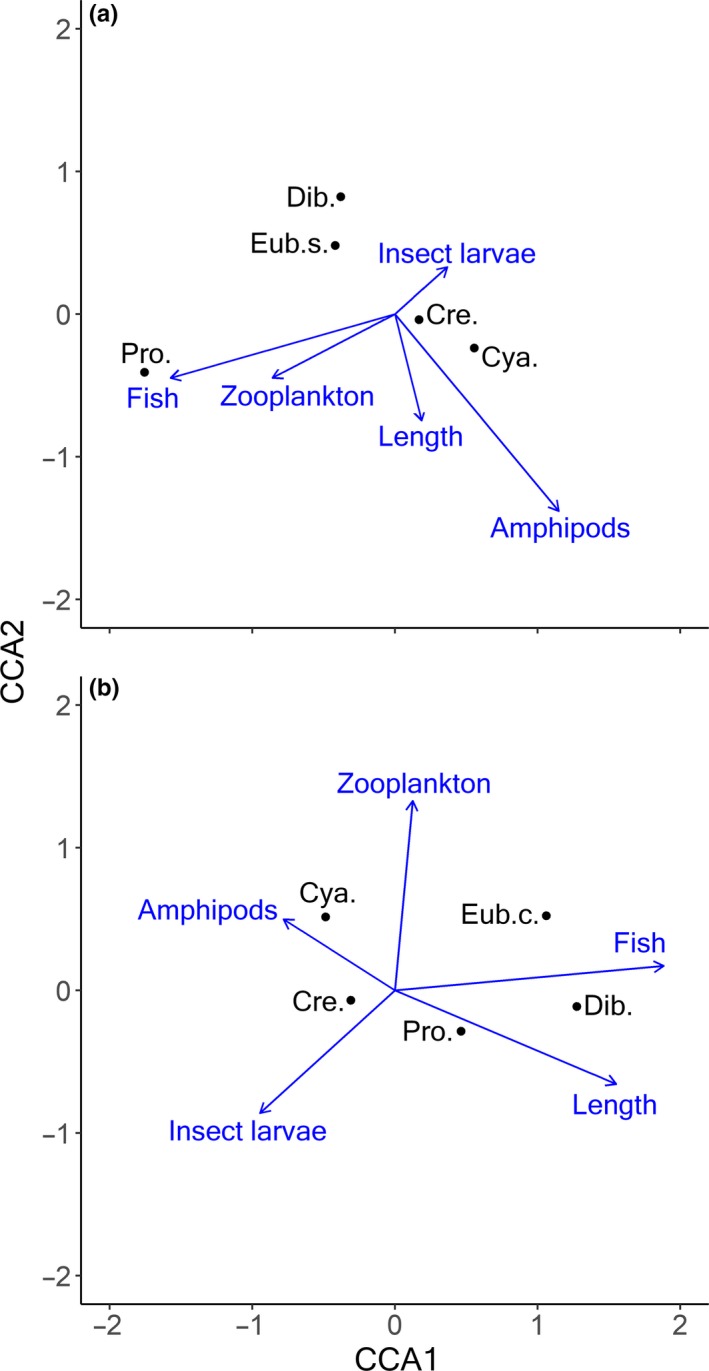
Canonical correspondence analysis (CCA) performed on parasite abundances as a function of presence‐absence of prey types and fish length in (a) Arctic charr and (b) brown trout. (Cre. = *Crepidostomum* spp., Cya. = *C. truncatus,* Eub.s. = *E. salvelini*, Eub.c. = *E. crassum*, Pro. = *Proteocephalus* sp., and Dib. = *Dibothriocephalus* spp.)

In Arctic charr, host length and the frequency of occurrence of amphipods, insect larvae, zooplankton, and fish in the diet, were all significantly associated with variation in parasite abundance (CCA; permutation test, all *p* < .05). Together the first two dimensions of the CCA accounted for 21.6% of the total variation (Figure 6a). Dimension 1 was mostly correlated with the explanatory variables fish and zooplankton prey, which accounted for 18.1% of the total variation in the parasite abundance data. Dimension 2 was mostly correlated with amphipods prey and accounted for 3.5% of the total variation (Figure 6a). In brown trout, host length and the frequency of occurrence of fish prey and amphipods in the diet were significantly associated with the variation in parasite abundance data (CCA; permutation test, all *p* < .05), whereas the frequencies of insect larvae and zooplankton were not significant. The first two dimensions explained 19.9% of the total variation (Figure 6b). Dimension 1, which accounted for 17.4% of the total variation, was mostly correlated with fish prey in the diet and host length, while dimension 2 was driven mainly by predation on zooplankton and explained 2.5% of the total variation (Figure 6b).

Similar patterns were observed between parasite prevalence and frequency of occurrence of prey types in the diet of both Arctic charr and brown trout. In Arctic charr, the prevalence of both *C. truncatus* (rw = 0.85, *p* < .05) and *Crepidostomum* spp. (rw = 0.71, *p* = .067) was positively correlated with the frequency of occurrence of amphipods in the diet, whereas *Proteocephalus* sp. was highly correlated with zooplankton (rw = 0.91, *p* < .01) and fish prey (rw = 0.86, *p* < .05). Other diet‐parasite correlations were not significant. In brown trout, the prevalence of *Dibothriocephalus* spp. (rw = 0.98, *p* < .01) was highly correlated with the frequency of occurrence of fish prey. The prevalence of *Proteocephalus* sp. was highly correlated with the occurrence of amphipods in the diet (rw = 0.90, *p* < .05), even though this is not an intermediate host. Other diet‐parasite correlations were not significant.

**Figure 7:**
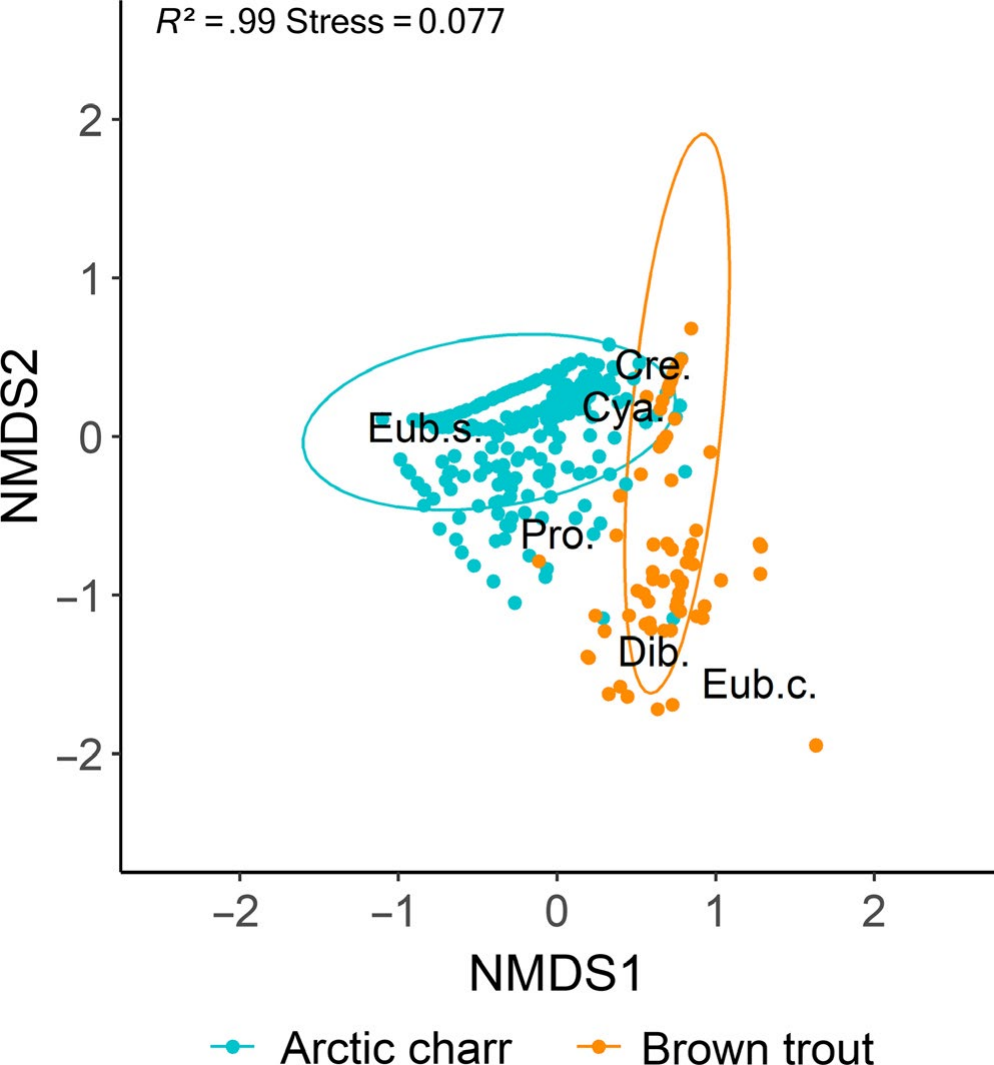
Differences in parasite community composition between Arctic charr and brown trout using nonmetric multidimensional scaling (NMDS) plot on Bray–Curtis distances including 95% confidence interval ellipses (Cre. = *Crepidostomum* spp., Cya. = *C. truncatus*, Eub.s. = *E. salvelini*, Eub.c. = *E. crassum*, Pro. = *Proteocephalus* sp., and Dib. = *Dibothriocephalus* spp.). NMDS converged on a three‐dimensional solution with an acceptable stress level

Overall, the parasite communities of Arctic charr and brown trout were segregated (Figure 7). In Arctic charr, the elevated abundance of *C. truncatus* and *Crepidostomum* spp. was associated with amphipod consumption, while *Proteocephalus* sp. was associated with fish and zooplankton prey groups. Brown trout consumed more fish and consequently had a higher abundance of *Dibothriocephalus* spp.

## 4 DISCUSSION

Our study revealed distinct seasonal patterns in the prevalence of several intestinal parasite taxa leading to temporal shifts in the parasite community composition in both Arctic charr and brown trout. The observed seasonality in parasite infections also underlines the significance of the winter season as an important transmission window for certain trophically transmitted parasite taxa because dietary shifts related to prey availability occurred in both salmonid species. The diet niche of Arctic charr and brown trout differed in all seasons, and this was mirrored in the structure of their parasite communities. The more opportunistic feeding behavior observed in Arctic charr apparently increased its exposure to trophically transferred parasites. In accordance with our first hypothesis, Arctic charr not only hosted more parasite taxa but also had an overall higher abundance and prevalence of helminths compared with brown trout. Hence, the host with the broadest niche (Arctic charr) harbored the richest community of food‐transmitted parasites, which is also in agreement with the expectations proposed by Kennedy, Bush, and Aho (1986) and Holmes (1990). Similar patterns have been observed in terrestrial animals. A study conducted on six sympatric species of lemurs in Kirindy Forest (Madagascar) showed that the mouse lemur (*Microcebus murinus*), which possessed the widest trophic niche, also had the highest burden of gastrointestinal parasites (Springer & Kappeler, 2016).

Among the four shared intestinal parasites, *Crepidostomum* spp., *C. truncatus,* and *Proteocephalus* sp. were more common in Arctic charr, while *Dibothriocephalus* spp. was more common in brown trout. Arctic charr feed more on zooplankton and amphipods compared with brown trout. Zooplankton includes copepods which are known to transmit *Eubothrium* spp., *Dibothriocephalus* spp., and *Proteocephalus* sp. (Knudsen et al., 2008; Kuhn, Knudsen, et al., 2016), while the amphipod *G. lacustris* is known to transmit several parasites including *C. truncatus* and *Crepidostomum* spp. (Hoffman, 1999; Okaka, 1984; Thomas, 1958; Vik, 1958).

In accordance with our second hypothesis, that Arctic charr will have increased infections of amphipod‐transmitted parasites during the ice‐covered period, the prevalence and intensity of *C. truncatus* and *Crepidostomum* spp. reached a peak in Arctic charr in early winter. Their elevated infections are explained by an early winter peak in predation on their intermediate host, the amphipod *G. lacustris*, which is also in agreement with findings from a nearby lake (Amundsen & Knudsen, 2009; Amundsen, Knudsen, Kuris, & Kristoffersen, 2003; Knudsen et al., 2008). Furthermore, the establishment success and residence time of *C. truncatus* in the fish host seem to increase with temperatures lower than 10°C (Amundsen et al., 2003; Awachie, 1968). Hence, the low water temperatures in the ice‐bound period likely prolong the residence time in the fish host (Amundsen et al., 2003), resulting in increased infection levels.

In brown trout, on the other hand, a distinct peak in prevalence of *Dibothriocephalus* spp. and *E. crassum* was observed in late winter coinciding with a profound rise in piscivory. The copepod‐transmitted helminths *Dibothriocephalus* spp. and *E. crassum* do have the ability to re‐establish in piscivorous fish (Von Bonsdorff & Bylund, 1982; Bylund, 1969; Halvorsen, 1970; Vik, 1963; Williams & Jones, 1994). Given the low frequency of occurrence of zooplankton in the diet of brown trout, piscivory appears to be important for transmission of these parasite taxa through reinfection processes, in particular by eating three‐spined sticklebacks. This assumption is further supported by the fact that *Dibothriocephalus* spp. was solely present as unencysted plerocercoids in the intestines of both Arctic charr and brown trout; and not as procercoids as should have been the case if they had been transmitted through ingestion of copepods. Previous studies conducted in Lake Takvatn, have highlighted the importance of stickleback as paratenic hosts in transmitting parasite to brown trout (Amundsen et al., 2013; Henriksen et al., 2016; Klemetsen et al., 2002; Kuhn, Amundsen, Kristoffersen, Frainer, & Knudsen, 2016). Similarly, a study of the brackish water of the Bothnian Bay, which included the winter period, found that the predatory burbot (*Lota lota*) was infected by *Diphyllobotrium ditremum* and *Eubothrium rugosum* through fish consumption (Valtonen & Julkunen, 1995). The present study shows that a substantial portion of this transmission probably occurs under ice cover in winter, which is also seen in other host‐parasites systems (Karvonen et al., 2005; Valtonen & Crompton, 1990; Valtonen et al., 1990).

In accordance with the third hypothesis, the accumulation of parasites through the winter period proved to be important for the differences in the overall parasite community seen between Arctic charr and brown trout. This pattern was to a great extent explained by seasonal changes in resource availability and feeding behavior between the two host species. Seasonal changes in parasite transmission to fish driven by intermediate host‐prey availability have also been observed in freshwater and coastal systems of tropical regions with contrasting dry/rain seasonal regimes (Moravec, Mendoza‐Franco, Vivas‐Rodríguez, Vargas‐Vázquez, & González‐Solís, 2002; Violante‐González, Aguirre‐Macedo, Rojas‐Herrera, & Guerrero, 2009; Violante‐González, Aguirre‐Macedo, & Vidal‐Martínez, 2008). In Arctic charr, the prevalence of the copepod‐transmitted parasite *Proteocephalus* sp. peaked in autumn and strongly declined during winter while the prevalence of amphipod‐transmitted parasites, notably *C. truncatus,* was high during the ice‐covered period. These temporal changes in the helminth communities of Arctic charr were well reflected by a corresponding dietary shift from zooplankton to benthic invertebrate, as most cladocerans enter the winter egg diapause and zooplankton availability thus decreases (Amundsen & Knudsen, 2009; Klemetsen et al., 2002; Klemetsen, Knudsen, et al., 2003). A reduction in zooplankton feeding during winter will consequently diminish the exposure to *Proteocephalus* sp., whereas and increase in consuming larger and more abundant zoobenthos favors the transmission of helminths like *Crepidostomum* spp. and *C. truncatus* that use amphipods and insect larvae as intermediate hosts. Reduced infections of *Proteocephalus longicollis* in common whitefish (*Coregonus lavaretus*) during the winter period following a decrease in zooplankton availability were also observed in a French perialpine lake by Hanzelová and Gerdeaux (2003). Increased infections of *Crepidostomum metoecus* and *C. truncatus* due to amphipod predation were also previously observed in Dolly Varden (*Salvelinus malma*) from Lake Dal'nee, Kamchatka (Busarova, Esin, Butorina, Esipov, & Markevich, 2017). A reduced exposure to copepod‐transmitted parasites during winter resulted in decreased diversity in the intestinal parasite community of Arctic charr in Lake Takvatn. Furthermore, the prevalence of *Crepidostomum* spp., *E. salvelini,* and *Dibothriocephalus* spp. did not show marked seasonal patterns in Arctic charr. The taxa *Crepidostomum* spp. comprise at least four different species that are transmitted thought insect larvae and/or amphipods (Soldánová et al., 2017). If some species of *Crepidostomum* only use insect larvae as intermediate host (see Marcogliese, Goater, & Esch, 1990), these may have higher prevalence during summer when the feeding on insect prey is high, while the prevalence of those transmitted through amphipods may increase toward winter, consequently explaining the lack of seasonality in *Crepidostomum* ssp. harbored by Arctic charr. The stability in the infections of *E. salvelini* suggests that the prevalence of this parasite is less affected by seasonal changes in diet due to a life span of more than 1 year in the fish host (Hanzelová, Scholz, Gerdeaux, & Kuchta, 2002; Hernandez & Muzzall, 1998). The low and stable prevalence of *Dibothriocephalus* spp. coincided with a low inclusion of fish prey in the diet of Arctic charr.

Similar to Arctic charr, distinct parasite‐diet relationships were also found in brown trout. The prevalence of parasites potentially transmitted by fish prey (i.e., *Dibothriocephalus* spp. and *E. crassum*) increased through the winter in correspondence with a transition from an insect‐dominated diet during summer to a more fish‐dominated diet in late winter. This shift in diet also explains a rise in the overall diversity of the intestinal parasite community of brown trout. The high degree of piscivory observed in trout during the winter period might partially relate to increased competition for benthic resources with Arctic charr (Amundsen & Knudsen, 2009). The modest winter sample of brown trout may on the other hand also be biased by an overrepresentation of larger fish, which might have contributed both to the higher degree of piscivory and the enhanced intensity of *Dibothriocephalus* spp. plerocercoids observed during winter. A peak in prevalence of *C. truncatus* between autumn and early winter was also observed, corresponding to a higher frequency of occurrence of amphipods in the autumn diet of brown trout. Not all brown trout parasites showed marked seasonality as the prevalence of *Crepidostomum* spp. remained high in all seasons. This is not surprising, given that brown trout exhibited persistent feeding on insect larvae throughout the year. *Crepidostomum* spp. use both insect larvae (Ephemeroptera) and the amphipod *G. lacustris* as intermediate hosts and might be able to survive in the fish‐host intestine up to 1 year (Curtis et al., 1995; Knudsen, Kristoffersen, & Amundsen, 1997; Kristmundsson & Richter, 2009; Thomas, 1958). Overall, the intestinal parasite communities of both Arctic charr and brown trout changed distinctly throughout the year and were associated with seasonal dietary shifts, with the winter period playing an important role for parasite transmissions. Interspecific interactions among the intestinal parasites might also have influenced the observed patterns, but such interactions have not been reported as important structuring forces for these taxa (Kuhn, Knudsen, et al., 2016).

In conclusion, our study reveals that the long‐lasting ice‐covered period represents an important transmission window for parasites to fish in high‐latitude lakes. Enhanced infections of amphipod‐transmitted parasites in Arctic charr and piscivore‐related parasites in brown trout during the ice‐cover period are chiefly explained by their winter diet. In essence, our findings document that the intestinal parasite communities of Arctic charr and brown trout are highly influenced by their seasonal dietary changes.

## CONFLICT OF INTEREST

The authors declare that they have no conflict of interest.

## AUTHOR CONTRIBUTIONS

Sebastian Prati, Eirik H. Henriksen, Rune Knudsen, and Per‐Arne Amundsen conceived the idea and designed the methodology; Sebastian Prati, Eirik H. Henriksen, and Per‐Arne Amundsen conducted fieldwork; Sebastian Prati analyzed the data; Sebastian Prati led the writing on the manuscript with additional contributions from Eirik H. Henriksen, Rune Knudsen, and Per‐Arne Amundsen. All authors contributed critically to the drafts and gave final approval for publication. **Sebastian Prati**: Conceptualization (equal); data curation (lead); formal analysis (lead); investigation (equal); methodology (equal); writing‐original draft (lead); writing‐review and editing (lead). **Eirik H. Henriksen**: Conceptualization (equal); investigation (equal); methodology (equal); supervision (supporting); writing‐original draft (supporting). **Rune Knudsen**: Conceptualization (equal); methodology (equal); supervision (supporting); writing‐original draft (supporting). **Per‐Arne Amundsen**: Conceptualization (equal); investigation (equal); methodology (equal); supervision (lead); writing‐original draft (supporting).

## ETHICAL APPROVAL

All applicable institutional and/or national guidelines for the care and use of animals were followed.

## Data Availability

Raw data associated with this paper are available on Dryad, https://doi.org/10.5061/dryad.r2280gb8w
